# Dermatological Features as a Diagnostic Clue in Systemic Loxocelism Caused by Brown Recluse Spider Bite

**DOI:** 10.4269/ajtmh.23-0246

**Published:** 2023-09-18

**Authors:** Natalia Isabel Suárez-Ospino, Edier Díaz-Anaya, Andrés Felipe Ochoa-Díaz

**Affiliations:** ^1^ School of Medicine, Universidad de Santander, Bucaramanga, Colombia;; ^2^ School of Medicine, Universidad Cooperativa de Colombia, Bucaramanga, Colombia;; ^3^ Internal Medicine Department, Universidad Industrial de Santander, Bucaramanga, Colombia

**Figure 1.**
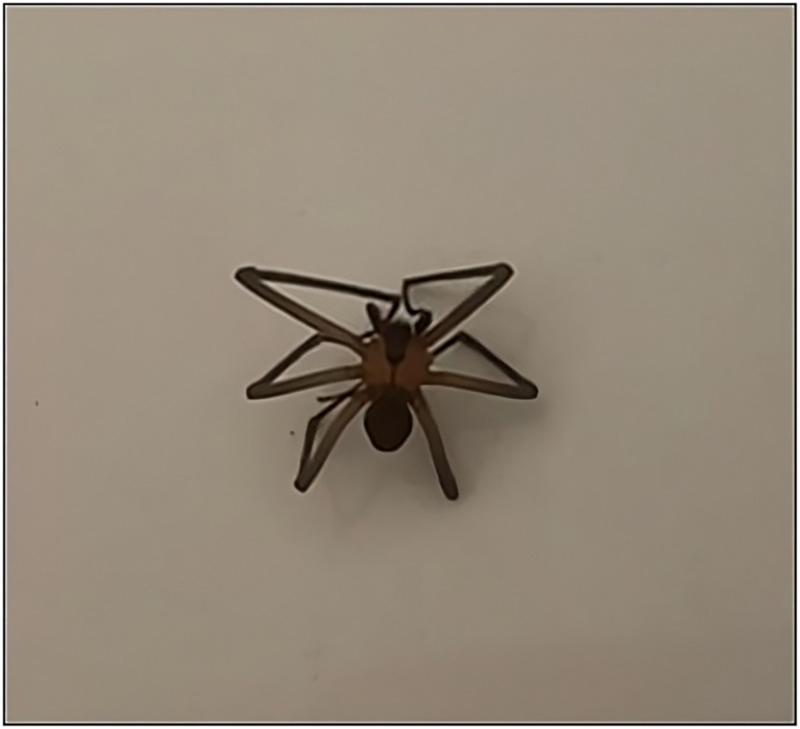
Photo of the brown recluse spider provided by the patient.

**Figure 2.**
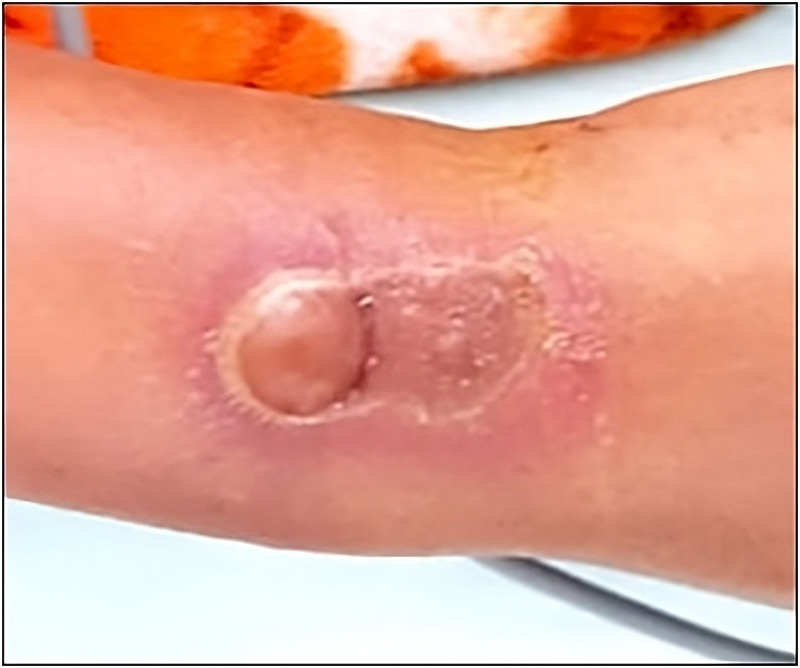
Right wrist flat plaque with central pale, erythematous edges and proximal small blister at site of spider bite.

**Figure 3.**
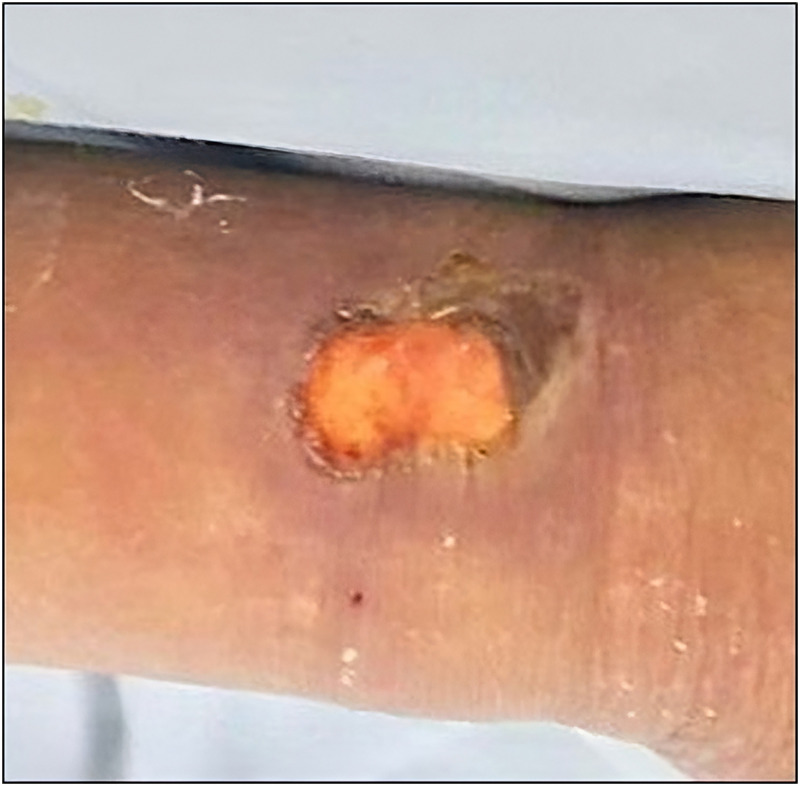
Right wrist ulcer with clean bottom, regular and erythematous edges, and ruptured blister 10 days after spider bite.

The patient, a 26-year-old woman from northeast Colombia, presented with a painless lesion on her right wrist after a spider bite at home. She described the animal as a < 5 cm “violin spider” (Figure 1). She presented to the hospital 5 days later with severe shortness of breath, lower limb edema, malaise, and oliguria. A single plaque on the right wrist was present (Figure 2). Laboratory tests showed leukocytosis (15,520/μL) with neutrophilia, hemoglobin of 9 g/dL, normal platelet count, elevated of C-reactive protein (67.4 mg/dL), alanine aminotransferase of 215 U/L, aspartate aminotransferase of 117 U/L, and conjugated hyperbilirubinemia 4.2 mg/dL. There was severe metabolic acidemia, acute kidney injury with elevated serum creatinine (18 mg/dL), and blood urea nitrogen (125 mg/dL). Tests of coagulation and hemolysis were normal. Hypoxemic respiratory failure developed, requiring mechanical ventilation; hemodialysis was begun. The history of spider bite, typical cutaneous lesion, and organ dysfunction led to the diagnosis of systemic loxocelism. The cutaneous lesion on right wrist progressed to an ulcer with regular edges and clean bottom, with persistence of erythematous halo and ruptured blister after 4 days (Figure 3). She was not a candidate for specific antidote due to time of evolution at presentation according to national toxicology guidelines. Progressive multiorgan failure led to death 10 days after admission.

Spiders of the genus *Loxoceles* are small arthropods of 1 to 3 cm in size that typically live in dry, dark places such as furniture and clothes in the home environment. Bites by this spider occur when they are pressed against human body, causing signs and symptoms known as loxocelism. Cutaneous involvement is reported in 83% of patients bitten; systemic compromise is less frequent, with acute kidney injury seen in 14.2% of cases.^1^^–^^3^

*Loxoceles* bite lesions are unique and usually follow the spider’s defensive response to close contact. *Loxoceles* spider venom contains phospholipase D, hyaluronidases, and metalloproteases that damage vascular endothelial cells,^4^ the consequences of which lead to dermonecrotic manifestations and systemic complications typical of those described here.^1^ The lesions have a pale center and are flat or slightly raised. Their size does not exceed 10 cm; they appear with ulcerations after 7 to 14 days and are accompanied by small blisters with clear serous material,^5^ as seen in the patient described here.

Recognizing the characteristics of cutaneous loxocelism is important to accelerate timely antivenom treatment to forestall systemic consequences, especially in patients with progressive organ dysfunction and history of spider bite.
